# Dimethylarginines in Children after Anti-Neoplastic Treatment

**DOI:** 10.3390/medicina58010108

**Published:** 2022-01-11

**Authors:** Michalina Jezierska, Anna Owczarzak, Joanna Stefanowicz

**Affiliations:** 1 Department of Paediatrics, Haematology and Oncology, Faculty of Medicine, Medical University of Gdansk, 7 Debinki Street, 80-211 Gdansk, Poland; michalina.jezierska@gumed.edu.pl; 2 Department of Paediatrics, Haematology and Oncology, University Clinical Centre, 7 Debinki Street, 80-952 Gdansk, Poland; 3 Department of Clinical Nutrition and Dietetics, Faculty of Health Sciences with Institute of Maritime and Tropical Medicine, Medical University of Gdansk, 7 Debinki Street, 80-211 Gdansk, Poland; annaow@gumed.edu.pl; 4 Faculty of Health Sciences, Medical University of Gdansk, 7 Debinki Street, 80-211 Gdansk, Poland

**Keywords:** leukaemia, solid tumours, survivors, late effects, nephrotoxicity, dimethylarginines

## Abstract

*Background and Objectives*: According to a recent Cochrane systematic review, renal impairment can develop in 0–84% of childhood cancer survivors in the future. The renal function impairment in this patient group can be related to nephrectomy, nephrotoxic agents therapy, abdominal radiotherapy, and combinations of these treatment methods. In this study, in a population of patients after anti-neoplastic therapy, with particular emphasis on patients after Wilms’ tumour treatment, we compared new substances which play role in the chronic kidney disease (CKD) pathogenesis (asymmetric dimethylarginine—ADMA, symmetric dimethylarginine—SDMA) with standard renal function markers (e.g., creatinine and cystatin C in serum, creatinine in urine, etc.) to assess the usefulness of the former. *Materials and Methods*: Eighty-four children, without CKD, bilateral kidney tumours, congenital kidney defects, or urinary tract infections, with a minimum time of 1 year after ending anti-neoplastic treatment, aged between 17 and 215 months, were divided into three groups: group 1—patients after nephroblastoma treatment (*n* = 21), group 2—after other solid tumours treatment (*n* = 44), and group 3—after lymphoproliferative neoplasms treatment (*n* = 19). The patients’ medical histories were taken and physical examinations were performed. Concentrations of blood urea nitrogen (BUN), creatinine, cystatin C, C-reactive protein (CRP), ADMA, and SDMA in blood and albumin in urine were measured, and a general urine analysis was performed. The SDMA/ADMA ratio, albumin–creatine ratio, and estimated glomerular filtration rate (eGFR) were calculated. eGFR was estimated by three equations recommended to the paediatric population by the KDIGO from 2012: the Schwartz equation (eGFR1), equation with creatinine and urea nitrogen (eGFR2), and equation with cystatin C (eGFR3). *Results*: Both the eGFR1 and eGFR2 values were significantly lower in group 1 than in group 3 (eGFR1: 93.3 (83.1–102.3) vs. 116.5 (96.8–126.9) mL/min/1.73 m^2^, *p* = 0.02; eGFR2: 82.7 (±14.4) vs. 94.4 (±11.9) mL/min/1.73 m^2^, *p* = 0.02). Additionally, there were weak positive correlations between SDMA and creatinine (*p* < 0.05, r = 0.24), and cystatin C (*p* < 0.05, r = 0.32) and weak negative correlations between SDMA and eGFR1 (*p* < 0.05, r = −0.25), eGFR2 (*p* < 0.05, r = −0.24), and eGFR3 (*p* < 0.05, r = −0.32). *Conclusions*: The usefulness of ADMA and SDMA in the diagnosis of renal functional impairment should be assessed in further studies. eGFR, calculated according to equations recommended for children, should be used in routine paediatric practice.

## 1. Introduction

Significant progress has been made in paediatric oncology in recent decades. Nearly 80% of children treated for neoplasms are cured or remain in long-term remission [1,2]. The growing population of people who have received anti-neoplastic therapy in childhood is a natural consequence of this success. This population needs further medical supervision due to the risk of complications after completed treatment. Renal impairment in the future can develop in 0–84% of survivors according to a recent Cochrane systematic review [3]. Such divergent data are an effect of individual differences in the performed diagnosis, anti-neoplastic therapy, and supportive care [1,4]. According to the Childhood Cancer Survivors Study, 0.5% of childhood cancer survivors develop renal failure, and the mean age and mean time from initial diagnosis are 27 and 18 years, respectively [5]. Patients treated with ifosfamide and cisplatin after nephrectomy are especially prone to having kidney damage [4,6].

Wilms tumour is the most common kidney neoplasm in children. It is also one of the causes of CKD in children, although it is listed at the end of such reports. These two statistics are connected by the word kidney, but Wilms tumour means much more: nephrectomy (unique among neoplasm treatment methods), nephrotoxic agents, abdominal radiotherapy, and their combinations. Approximately 1% of patients develop end-stage renal disease (ESRD) after Wilms tumour treatment. When tumours occur bilaterally or are connected with WAGR and Denys–Drash syndromes, this value is higher, but we excluded such situations in our research [7,8]. Schiavetti et al., in a study of unilateral non-syndromic renal tumour survivors, confirmed that only a small portion of childhood cancer survivors developed CKD after two decades, but at the same time they found that many more survivors (22.9%) presented with a mild decrease in eGFR [9]. Green et al. confirmed that other complications, such as hypertension, are reported in this group of patients more often than ESRD [10]. The importance of these findings for the development of CKD is uncertain and can be dependent on other comorbid factors, such as hypertension or albuminuria. The coincidence with age-dependent changes in eGFR may also be important, as described by Cozzi et al. in research on the adaptation of kidney function after the treatment of children with unilateral kidney tumours [11].

Although risk factors for nephrotoxicity are quite well known, the process itself is complicated and can develop by various mechanisms. The estimated glomerular filtration rate (eGFR), recommended in the paediatric population for renal functional assessment, illustrates the accumulation of only some uraemic toxins, which can play an important role in the development of chronic kidney disease (CKD) and its complications [12,13,14]. Therefore, it seems to be necessary to seek new substances that can enhance our knowledge about nephrotoxicity and be clinically useful, providing a chance for early intervention and protection against kidney damage, as in, for example, the study of Stefanowicz et al. [15].

Asymmetric dimethylarginines (ADMA) and symmetric dimethylarginines (SDMA), which are post-translational derivatives of arginine-containing proteins, and nitric oxide production inhibitors may be important components of the deterioration of renal function and of the puzzle connecting chronic kidney disease to cardiovascular diseases [16,17,18,19]. There are reports that a higher concentration of ADMA was connected with a faster loss of renal function, development of hypertension, and higher mortality in patients with CKD [20,21]. Its optical isomer, SDMA, as a substance that is eliminated mainly by urine excretion, correlates with eGFR and creatinine clearance [16,20,21]. Both substances are independent risk factors for all mortality and cardiovascular disease [21].

The aim of this study was to assess the usefulness of ADMA and SDMA concentrations in blood in the early diagnosis of impaired renal function in children after anti-neoplastic treatment, excluding patients with CKD, bilateral kidney tumours, congenital kidney defects, or urinary tract infections.

## 2. Materials and Methods

### 2.1. Patient Selection and Characteristics

Eighty-four children, without CKD, bilateral kidney tumours, congenital kidney defects, or urinary tract infections, who ended anti-neoplastic treatment a minimum of 1 year ago, were included in the study. All of these children were patients of the Department of Paediatrics, Haematology and Oncology Medical University of Gdansk, Clinical University Centre in Gdansk. The study inclusion and exclusion criteria are presented in Table 1.

The study population was aged between 17 and 215 months, with the median age and IQR of 134.5 (77.5 ÷ 173) months. There were 49 boys and 35 girls (1.4:1/58%:42%). The study population was divided into three groups: group 1—patients after Wilms tumour treatment (*n* = 21); group 2—patients after other solid tumour treatments (*n* = 44, neuroblastomas—19, rhabdomyosarcomas—9, hepatoblastomas—6, brain tumours—5, germ cell tumours—3, osteosarcoma—1, Ewing sarcoma—1); and group 3—patients after lymphoproliferative neoplasm treatment (*n* = 19). During a routine visit in the department, the patients’ medical histories were taken, and physical examinations with measurements of body mass, height, and blood pressure as well as laboratory tests were performed. Concentrations of BUN, creatinine, cystatin C, C-reactive protein, ADMA, and SDMA were measured in a single blood sample, and creatinine and albumin were measured in a single urine sample. The SDMA/ADMA ratio, albumin–creatine ratio (ACR), and eGFR were also calculated.

### 2.2. Blood Pressure Measurements

Blood pressure (BP) was measured three times by means of the oscillometric technique. Then, the average values of systolic and diastolic pressure were calculated and referred to the reference standards for age, sex, and height.

### 2.3. Laboratory Tests

The following measurements were performed by the Central Clinical Laboratory of the University Clinical Centre in Gdansk: BUN, creatinine, cystatin C, and CRP determination in blood samples; creatinine and albumin determination in urine samples; and urine analysis. Creatinine was assessed with the immunoenzymatic method (Abbott Laboratories, Warsaw, Poland), cystatin C with the immunonephelometric method (Siemens Healthcare, Warsaw, Poland), BUN with the method with urease (Abbott Laboratories, Warsaw, Poland), and CRP and albumin with the turbidimetric method (Abbott Laboratories, Warsaw, Poland).

ADMA and SDMA determination in blood samples was performed by the Department of Clinical Nutrition Medical University of Gdansk. Immunoenzymatic methods with the ADMA ELISA KIT and SDMA ELISA KIT from Immunodiagnostic (Bensheim, Germany) were performed according to the manufacturer’s instructions.

### 2.4. eGFR Equations

eGFR was calculated according to formulas recommended for the paediatric population by The Kidney Disease: Improving Global Outcomes (KDIGO) from 2012 [12]:

(1) $$eGFR1=41.3\times \left(\frac{H}{creatinine_{s}}\right)$$

(2) $$eGFR2=40.7\times {\left(\frac{H}{creatinine_{s}}\right)}^{0.64}\times {\left(\frac{30}{BUN}\right)}^{0.202}$$

(3) $$eGFR3=70.69\times {\left(cystatin\right)}^{-0.931}$$

where *eGFR* is the estimated glomerular filtration rate (mL/min/1.73m^2^), *H* is the height (m), *creatinine_s_* is the serum creatinine level (mg/dL), *BUN* is the blood urea nitrogen (mg/dL), and *cystatin* is the serum cystatin C level (mg/L).

### 2.5. Statistical Analysis

The obtained results were analysed statistically. A normal distribution of the data was verified with the Shapiro–Wilk test. Parameters that followed normal distribution patterns are presented as the mean value ± SD, while parameters that were not normally distributed are presented as the median value and range of values. Parametric or nonparametric ANOVA tests were performed to compare groups, with the subsequent analysis of significant differences using the post hoc method. A correlation analysis was performed using Pearson’s or Spearman’s method, depending on the character and distribution of the correlated data. The level of significance was considered to be *p* < 0.05. Statistical analysis was performed using Dell Statistica software (Dell Inc.), version 13.

## 3. Results

The groups were similar in terms of age, sex, time from the end of treatment, body mass, height, systolic and diastolic blood pressure (SBP, DBP), and body mass index (BMI). The median time after the end of the anti-neoplastic treatment in our research was 56 months in group 1, 59 months in group 2, and 42.5 months in group 3. None of the studied patients met the clinical and laboratory criteria for the CKD diagnosis. The exact characteristics of the groups are presented in Table 2. The assessment of blood pressure (BP) is presented in Table 3. Table 4 shows the values of the assessed parameters obtained in each of the studied groups.

There were no statistically significant differences in the concentrations of ADMA and SDMA between the groups. There were no statistically significant differences in the concentrations of creatinine, BUN, or cystatin C in the blood, albumin in urine, or ACR between the groups. Differences in the values of eGFR calculated by any formula are shown between groups. The lowest eGFR value was observed in group 1, a higher value in group 2, and the highest value in group 3. However, statistically significant differences were observed only in eGFR_1_ and eGFR_2_ between group 1 and group 3. Both the median eGFR_1_ value and average eGFR_2_ value were significantly lower in group 1 than in group 3: eGFR_1_ 93.3 (83.1–102.3) vs. 116.5 (96.8–126.9) mL/min/1.73 m^2^, *p* = 0.02; eGFR_2_ 82.7 (±14.4) vs. 94.4 ±11.9 mL/min/1.73 m^2^, *p* = 0.02.

**Figure 1:**
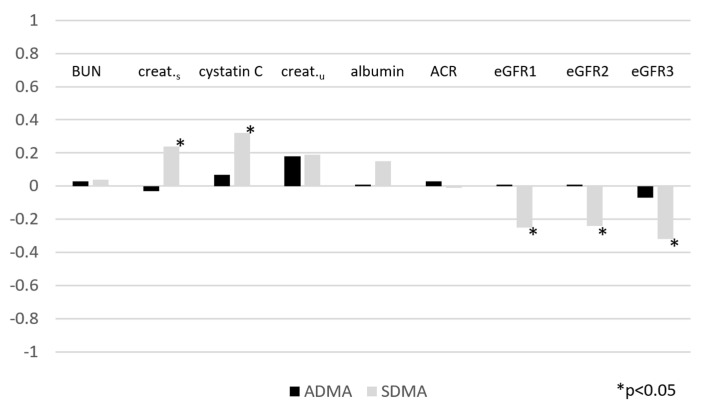
Spearman’s rank correlation coefficient for the studied parameters; ADMA-asymmetric dimethylarginine, SDMA-symmetric dimethylarginine, BUN-blood urea nitrogen, creat._s_-serum creatinine level, cystatin C-serum cystatin C level, creat._u_-urine creatinine level, ACR-albumin–creatine ratio, eGFR1-estimated glomerular filtration rate by the Schwartz equation, eGFR2-estimated glomerular filtration rate by equation with creatinine and urea nitrogen, eGFR3-estimated glomerular filtration rate by equation with cystatin C.

There were weak positive correlations between SDMA and creatinine (*p* < 0.05, r = 0.24) as well as cystatin C (*p* < 0.05, r = 0.32) and weak negative correlations between SDMA and eGFR_1_ (*p* < 0.05, r = −0.25), eGFR_2_ (*p* < 0.05, r = −0.24), and eGFR_3_ (*p* < 0.05, r = −0.32) in all patients. These results are documented in Figure 1. No other correlations between the assessed parameters were found.

## 4. Discussion

Six years have passed from the first review article, which concerns the assessment of renal function in people after anti-neoplastic treatment in childhood, until the last summary by the Cochrane Library [3,6]. Subsequent reports have since assured us that in children with oncological diseases, from the start of the diagnostic process until many years after the end of the treatment, renal function should be regularly assessed. Our research is another attempt to do so but with the additional use of new parameters (dimethylarginines) that play a role in the development and progression of CKD and cardiovascular risk connected with it. The main findings of our study are the observation that children treated for Wilms tumour have a lower eGFR than children treated for other neoplasms, especially lymphoproliferative diseases, and the existence of a correlation between SDMA and serum creatinine and cystatin C, which are used for the calculation of eGFR.

All our patients were under 18 years old. The time after the end of the anti-neoplastic treatment in our research was relatively short (see results). Based on the finding of Neu et al. of a correlation between a lower eGFR and a longer follow-up time among survivors of childhood Wilms tumours (mean age 28.7 years, mean follow-up 24.8 years), we can suppose that it would be worthwhile to extend our population observation time [22].

In our research, the groups showed no differences in ADMA, SDMA, creatinine, or cystatin C in blood; albumin in urine; or ACR. Differences appeared in eGFR values only, especially in eGFR based on serum creatinine. Serum creatinine has wide individual variability and is sex-, age-, and muscle mass-dependent [23,24]. Our study groups were similar in terms of age, sex, body mass percentile (cc), and height percentile (cc). Nevertheless, we cannot forget that as a result of disease and treatment, patients are burdened with body composition changes, especially disturbances in the proportions of fat and muscle mass [25]. Additionally, the method of creatinine determination is not without significance. According to KIDGO, it should be calibrated to the international standard reference, and bias compared to the reference method (isotope dilution mass spectrometry—IDMS) should be minimal. The enzymatic method used in our research seems to fulfil the conditions mentioned above, in contrast to the colorimetric method [12]. Cystatin C is almost exclusively glomerular filtration-dependent, but changes in its level are observed in neoplasms and during steroid therapy, which is one of the treatment methods for neoplasms, such as acute leukaemias [26]. In conclusion, neither creatinine nor cystatin C seem to be the best parameter for renal functional assessment in oncological patients, in contrast to adult patients as demonstrated by Vermassen et al. [27]. The earliest marker of deteriorating renal function appears to be eGFR, which was shown by Greer et al., who studied the effect of using eGFR on the timing of patient referral to specialist care by primary care physicians [28]. Decreased eGFR is still one of the CKD diagnostic criteria, and its value determines CKD stage classification [12]. The superiority of eGFR over the serum level of single substances such as creatinine or cystatin C fully explains why we observed only eGFR changes in our research. The fact that a lower eGFR value concerns Wilms tumour survivors is compatible with our primary thesis that this group has a higher risk of nephrotoxicity.

In our research, there were weak positive correlations between SDMA and creatinine (*p* < 0.05, r = 0.24) as well as cystatin C (*p* < 0.05, r = 0.32) and weak negative correlations between SDMA and eGFR_1_ (*p* < 0.05, r = −0.25), eGFR_2_ (*p* < 0.05, r = −0.24), and eGFR_3_ (*p* < 0.05, r = −0.32) in all patients. Our results are in line with the results of Jaźwińska-Kozuba et al., who also proved correlations of SDMA with creatinine and eGFR (r = 0.31, *p* = 0.05; r = −0.35, *p* = 0.03) in healthy children and adolescents [29]. They also demonstrated a superior SDMA/ADMA ratio over SDMA as a renal function parameter. Likewise, correlations between SDMA and creatinine and eGFR were found by Wasilewska et al., who compared children with chronic kidney disease stages 1–5 with a healthy control group. The correlation concerned both groups (renal dysfunction group in the following order: r = 0.56, *p* < 0.01; r = −0.65, *p* < 0.01; total subjects in the following order: r = 0.64, *p* < 0.01, r = −0.81, *p* < 0.01). Similar results that were related to SDMA and eGFR in a group of children with CKD were obtained by Snauwaert et al. (r = −0.82, *p* < 0.05), El-Sadek et al. (r = −0.692, *p* = 0.0007), and Brooks et al. (r = −0.74, *p* = 2.3 × 10^−7^), in a group of children with a solitary functioning kidney by Taranta-Janusz et al. (r = −0.409, *p* < 0.001), in a group of children with hypertension by Goonaskera et al. (r = −0.38, *p* < 0.002), in a group of children with diabetes type 1 by Marcovevvhio et al. 1 (r = −0.38, *p* < 0.001), and in a group of children born with low birth weight by Protas et al. (r = −0.39; *p* = 0.05) [14,30,31,32,33,34,35,36]. Paediatric population reports are supported by many more studies on adults, and one of many examples would be the Kilstein et al. study and their meta-analysis [37,38]. Different populations were included in the abovementioned studies. There are very few reports of methylarginines in patients treated in childhood because of neoplasms. In this respect our study is innovative. Except in the study by Snauwaert et al., the creatinine value was determined by the Jaffe method to be less accurate than the recommended enzymatic method. Most researchers limited themselves to using the most popular Schwartz equitation. Moreover, Saldek et al. used the MDRD equation, which may be questionable in paediatrics. We are the only ones to assess the GFR according to all equations recommended for the paediatric population, which is an unquestionable advantage of our study and opens up new opportunities for future investigators. Finally, the determination of SDMA took place using two different possibilities: ELISA and HPLC-MS/MS, which correlate moderately with each other. All of this makes it difficult to compare these studies directly.

The determination of creatinine concentration by the enzymatic method and comparison of the eGFR values calculated based on three different but recommended paediatric population formulas are the undoubted advantages of our study. However, there are also some limitations of our study, such as the relatively small study population, short time from the end of anti-neoplastic treatment to our study, the use of only single measurements of studied parameters, and the determination of ADMA and SDMA with the enzyme immunoassay method [16].

## 5. Conclusions

The usefulness of ADMA and SDMA in the early diagnosis of renal functional impairment in children after anti-neoplastic treatment cannot be assessed based on our study. Further studies are needed, especially in patients with CKD diagnosis, where it will be possible to compare dimethylarginines with abnormal value standard renal function markers, e.g., elevated creatine or decreased eGFR. New biomarkers of renal function can enhance our knowledge about nephrotoxicity and be clinically useful, providing a chance for early intervention and protection against kidney damage.

To assess kidney function, single substances are not enough. eGFR, calculated according to the equation recommended for children, should be used in routine paediatric practice. Such evaluation should be regular and long-term analysed in patients at risk of developing impairment of renal function, for example, patients after Wilms tumour treatment.

## Figures and Tables

**Table 1:** The inclusion and exclusion criteria of the study.

Inclusion Criteria	Exclusion Criteria
Age < 18 years	Age < 1 year
Min. 1 year after anti-neoplastic treatment	Chronic kidney disease
	Bilateral kidney tumours
	Congenital kidney defects
	Urinary tract infections
	Pregnancy

**Table 2:** Age, time from the end of treatment, body weight, height, and body mass index (BMI) in the study groups.

Characteristics	Group 1	Group 2	Group 3	*p* Value
Age (months) *	146.0 (96.0–182.0)	126.0 (70.0–173.0)	147.0 (80.0–172.0)	0.49
End of treatment (months) *	56.0 (43.0–111.0)	59.0 (31.0–88.0)	42.5 (24.0–53.0)	0.08
Body mass (percentile) *	42.0 (15.3–71.0)	61.0 (21.0–88.5)	57.0 (22.0–85.0)	0.57
Height (percentile) *	51.0 (33.0–78.0)	44.0 (14.5–78.5)	54.0 (32.0–80.0)	0.62
BMI (percentile) *	41.0 (18.0–74.0)	54.0 (32.0–92.0)	58.0 (23.0–89.0)	0.33

* Results are presented as median and IQR.

**Table 3:** Blood pressure (BP) in the study groups.

BP	Group 1	Group 2	Group 3	*p* Value
SBP (percentile) *	53.0 (24.0–88.0)	60.0 (38.0–81.0)	63.5 (32.0–96.0)	0.78
DBP (percentile) *	84.0 (58.0–93.0)	81.0 (57.0–91.0)	74.0 (63.0–88.0)	0.88

* Results are presented as median and IQR. SBP—Systolic blood pressure, DBP—diastolic blood pressure.

**Table 4:** Renal assessment in the study groups.

Renal Function Parameter	Group 1	Group 2	Group 3	*p* Value
ADMA (µmol/L) *	2.5 (1.5–3.8)	2.4 (1.1–4.5)	3.3 (1.1–5.2)	0.90
SDMA (µmol/L) *	1.6 (1.3–1.9)	1.2 (1.0–2.6)	1.0 (0.8–3.7)	0.48
BUN (mg/dL) *	12.7 (10.4–16.1)	12.9 (10.4–15.6)	11.3 (10.1–13.3)	0.33
Creatinine_s_ (mg/dL) **	0.7 (±0.2)	0.6 (±0.2)	0.6 (±0.1)	0.12
Cystatin C (mg/L) **	0.8 (±0.1)	0.8 (±0.1)	0.7 (±0.1)	0.12
eGFR1 (mL/min/1.73 m^2^) *	93.3 (83.1–102.3)	101.6 (89.4–119.0)	116.5 (96.8–126.9)	0.02 ***
eGFR2 (mL/min/1.73 m^2^) **	82.7 (±14.4)	87.2 (±12.7)	94.4 (±11.9)	0.02 ***
eGFR3 (mL/min/1.73 m^2^) **	85.9 (±12.6)	89.1 (±15.0)	94.9 (±12.6)	0.13
Creatinine_u_ (mg/dL) *	74.7 (56.0–127.1)	68.8 (43.4–104.9)	75.6 (64.0–144.4)	0.57
Albumin (mg/L) *	16.3 (7.5–26.0)	13.6 (9.3–24.8)	15.5 (11.9–22.6)	0.91
ACR (mg/g creatinine) *	14.7 (9.3–21.9)	16.4 (11.2–53.9)	17.4 (8.2–36.0)	0.63

* Results are presented as median and IQR, ** results are presented as average ± SD, *** significance was set as *p* < 0.05; group 1—patients after nephroblastoma treatment, group 2—patients after other solid tumours treatment, group 3—patients after lymphoproliferative neoplasms treatment, ADMA—asymmetric dimethylarginine, SDMA—symmetric dimethylarginine, BUN—blood urea nitrogen, creatinine_s_—serum creatinine level, cystatin C—serum cystatin C level, eGFR1—estimated glomerular filtration rate by the Schwartz equation, eGFR2—estimated glomerular filtration rate by equation with creatinine and urea nitrogen, eGFR3—estimated glomerular filtration rate by equation with cystatin C, creatinine_u_—urine creatinine level, ACR—albumin–creatine ratio.

## Data Availability

The analyzed data sets generated during the study are available from the corresponding author on reasonable request for noncommercial use.

## References

[1] Krawczuk-Rybak M., Panasiuk A., Stachowicz-Stencel T., Zubowska M., Skalska-Sadowska J., Sęga-Pondel D., Czajńska-Deptuła A., Sławińska D., Badowska W., Kamieńska E. (2018). Health status of Polish children and adolescents after cancer treatment. Eur. J. Nucl. Med. Mol. Imaging.

[2] Krawczuk-Rybak M. (2013). Late effects of treatment of childhood cancer—On the basis of the literature and own experience. Med. Wieku Rozw..

[3] Kooijmans E.C., Bökenkamp A., Tjahjadi N.S., Tettero J.M., Broeder E.V.D.-D., Van Der Pal H.J., A Veening M. (2019). Early and late adverse renal effects after potentially nephrotoxic treatment for childhood cancer. Cochrane Database Syst. Rev..

[4] Skinner R. (2018). Late renal toxicity of treatment for childhood malignancy: Risk factors, long-term outcomes, and surveillance. Pediatr. Nephrol..

[5] Oeffinger K.C., Mertens A.C., Sklar C.A., Kawashima T., Hudson M.M., Meadows A.T., Friedman D.L., Marina N., Hobbie W., Kadan-Lottick N. (2006). Chronic Health Conditions in Adult Survivors of Childhood Cancer. N. Engl. J. Med..

[6] Mulder R.L., Knijnenburg S.L., Geskus R.B., Van Dalen E.C., Van Der Pal H.J.H., Koning C.C.E., Bouts A.H., Caron H.N., Kremer L.C.M. (2013). Glomerular Function Time Trends in Long-Term Survivors of Childhood Cancer: A Longitudinal Study. Cancer Epidemiol. Biomark. Prev..

[7] Breslow N.E., Collins A.J., Ritchey M.L., Grigoriev Y.A., Peterson S.M., Green D.M. (2005). End Stage Renal Disease in Patients with Wilms Tumor: Results from The National Wilms Tumor Study Group and The United States Renal Data System. J. Urol..

[8] Lange J., Peterson S.M., Takashima J.R., Grigoriev Y., Ritchey M.L., Shamberger R.C., Beckwith J.B., Perlman E., Green D.M., Breslow N.E. (2011). Risk Factors for End Stage Renal Disease in Non-WT1-Syndromic Wilms Tumor. J. Urol..

[9] Schiavetti A., Altavista P., De Luca L., Andreoli G., Megaro G., Versacci P. (2015). Long-term renal function in unilateral non-syndromic renal tumor survivors treated according to International Society of Pediatric Oncology protocols. Pediatr. Blood Cancer.

[10] Green D.M., Wang M., Krasin M.J., Davidoff A.M., Srivastava D., Jay D.W., Ness K.K., Shulkin B.L., Spunt S.L., Jones D.P. (2020). Long-term renal function after treatment for unilateral, nonsyndromic Wilms tumor. A report from the St. Jude Lifetime Cohort Study. Pediatr. Blood Cancer.

[11] Cozzi D.A., Ceccanti S., Frediani S., Mele E., Cozzi F. (2013). Renal function adaptation up to the fifth decade after treatment of children with unilateral renal tumor: A cross-sectional and longitudinal study. Pediatr. Blood Cancer.

[12] Levin A., Stevens P.E., Bilous R.W., Coresh J., De Francisco A.L.M., De Jong P.E., Griffith K.E., Hemmelgarn B.R., Iseki K., Lamb E.J. (2013). Kidney disease: Improving global outcomes (KDIGO) CKD work group. KDIGO 2012 clinical practice guideline for the evaluation and management of chronic kidney disease. Kidney Int. Suppl..

[13] Snauwaert E., Van Biesen W., Raes A., Glorieux G., Van Bogaert V., Van Hoeck K., Coppens M., Roels S., Walle J.V., Eloot S. (2018). Concentrations of representative uraemic toxins in a healthy versus non-dialysis chronic kidney disease paediatric population. Nephrol. Dial. Transplant..

[14] Snauwaert E., Van Biesen W., Raes A., Holvoet E., Glorieux G., Van Hoeck K., Van Dyck M., Godefroid N., Vanholder R., Roels S. (2018). Accumulation of uraemic toxins is reflected only partially by estimated GFR in paediatric patients with chronic kidney disease. Pediatr. Nephrol..

[15] Stefanowicz J., Owczuk R., Aleksandrowicz E., Owczarzak A., Kurylak A., Adamkiewicz-Drożyńska E., Balcerska A. (2012). Renal Function and Low–Molecular-Weight Proteins (Cystatin C, β2-Microglobulin, Neutrophil Gelatinase-associated Lipocalin) in Child and Young Adult Cancer Survivors. J. Pediatr. Hematol..

[16] Tain Y.-L., Hsu C.-N. (2017). Toxic Dimethylarginines: Asymmetric Dimethylarginine (ADMA) and Symmetric Dimethylarginine (SDMA). Toxins.

[17] Aldámiz-Echevarría L., Andrade F. (2012). Asymmetric Dimethylarginine, Endothelial Dysfunction and Renal Disease. Int. J. Mol. Sci..

[18] Hsu C.-N., Lu P.-C., Lo M.-H., Lin I.-C., Tain Y.-L. (2019). The Association between Nitric Oxide Pathway, Blood Pressure Abnormalities, and Cardiovascular Risk Profile in Pediatric Chronic Kidney Disease. Int. J. Mol. Sci..

[19] Roumeliotis S., Mallamaci F., Zoccali C. (2020). Endothelial Dysfunction in Chronic Kidney Disease, from Biology to Clinical Outcomes: A 2020 Update. J. Clin. Med..

[20] Rysz J., Gluba-Brzózka A., Franczyk B., Jabłonowski Z., Ciałkowska-Rysz A. (2017). Novel Biomarkers in the Diagnosis of Chronic Kidney Disease and the Prediction of Its Outcome. Int. J. Mol. Sci..

[21] Oliva-Damaso E., Oliva-Damaso N., Rodriguez-Esparragon F., Payan J., Baamonde-Laborda E., Gonzalez-Cabrera F., Santana-Estupiñan R., Rodriguez-Perez J.C. (2019). Asymmetric (ADMA) and Symmetric (SDMA) Dimethylarginines in Chronic Kidney Disease: A Clinical Approach. Int. J. Mol. Sci..

[22] Neu M.A., Russo A., Wingerter A., Alt F., Theruvath J., El Malki K., Kron B., Dittrich M., Lotz J., Stein R. (2017). Prospective analysis of long-term renal function in survivors of childhood Wilms tumor. Pediatr. Nephrol..

[23] Conkar S., Mir S., Karaslan F.N., Hakverdi G. (2018). Comparing different estimated glomerular filtration rate equations in assessing glomerular function in children based on creatinine and cystatin C. J. Clin. Lab. Anal..

[24] Den Bakker E., Gemke R., Van Wijk J.A.E., Hubeek I., Stoffel-Wagner B., Bökenkamp A. (2018). Combining GFR estimates from cystatin C and creatinine—What is the optimal mix?. Pediatr. Nephrol..

[25] Guolla L., Morrison K.M., Barr R.D. (2021). Adiposity in Survivors of Cancer in Childhood: How is it Measured and Why Does it Matter?. J. Pediatr. Hematol..

[26] Onopiuk A., Tokarzewicz A., Gorodkiewicz E. (2015). Cystatin C. A kidney function biomarker. Adv. Clin. Chem..

[27] Vermassen T., Geboes K., De Man M., Laurent S., Decoene E., Lumen N., Delanghe J., Rottey S. (2018). Neither creatinine- nor cystatin C-estimated glomerular filtration rate is optimal in oncology patients treated with targeted agents. Nephrol. Dial. Transplant..

[28] Greer R.C., Powe N.R., Jaar B.G., Troll M.U., Boulware L.E. (2011). Effect of primary care physicians’ use of estimated glomerular filtration rate on the timing of their subspecialty referral decisions. BMC Nephrol..

[29] Jaźwińska-Kozuba A., Martens-Lobenhoffer J., Surdacki A., Kruszelnicka O., Rycaj J., Godula-Stuglik U., Bode-Böger S.M. (2012). Associations between Endogenous Dimethylarginines and Renal Function in Healthy Children and Adolescents. Int. J. Mol. Sci..

[30] Wasilewska A., Taranta-Janusz K., Zoch-Zwierz W., Michaluk-Skutnik J. (2012). Is plasma symmetric dimethylarginine a suitable marker of renal function in children and adolescents?. Scand. J. Urol. Nephrol..

[31] El-Sadek A.E., Behery E.G., Azab A.A., Kamal N.M., Salama M.A., Abdulghany W.E., Abdallah E.A. (2016). Arginine dimethylation products in pediatric patients with chronic kidney disease. Ann. Med. Surg..

[32] Brooks E.R., Langman C.B., Wang S., Price H.E., Hodges A.L., Darling L., Yang A.Z., Smith F.A. (2009). Methylated arginine derivatives in children and adolescents with chronic kidney disease. Pediatr. Nephrol..

[33] Taranta-Janusz K., Wasilewska A., Stypułkowska J., Sutuła M. (2012). Osteopontin and symmetric dimethylarginine plasma levels in solitary functioning kidney in children. Acta Paediatr..

[34] Goonasekera C., Rees D.D., Woolard P., Frend A., Shah V., Dillon M.J. (1997). Nitric oxide synthase inhibitors and hypertension in children and adolescents. J. Hypertens..

[35] Marcovecchio M.L., Dalton R.N., Turner C., Prevost T., Widmer B., Amin R., Dunger P.D. (2010). Symmetric dimethylarginine, an endogenous marker of glomerular filtration rate, and the risk for microalbuminuria in young people with type 1 diabetes. Arch. Dis. Child..

[36] Protas P., Tenderenda-Banasiuk E., Taranta-Janusz K., Fiłonowicz R., Zając M., Wasilewska A. (2014). Is Symmetric Dimethylarginine a sensitive biomarker of subclinical kidney injury in children born with low birth weight?. Biomarkers.

[37] Kielstein J.T., Veldink H., Martens-Lobenhoffer J., Haller H., Burg M., Lorenzen J.M., Lichtinghagen R., Bode-Boger S.M., Kliem V. (2011). SDMA is an early marker of change in GFR after living-related kidney donation. Nephrol. Dial. Transplant..

[38] Kielstein J.T., Salpeter S.R., Bode-Boeger S.M., Cooke J., Fliser D. (2006). Symmetric dimethylarginine (SDMA) as endogenous marker of renal function—A meta-analysis. Nephrol. Dial. Transplant..

